# Accuracy of ultrasound in distinguishing pathology of malignant thyroid diseases: A protocol for systematic review and meta-analysis

**DOI:** 10.1097/MD.0000000000028556

**Published:** 2022-01-14

**Authors:** Ling Qin, Qiyu Liu, Lipeng Sun, Hui Wang

**Affiliations:** Ultrasound Department of the First Affiliated Hospital of Dalian Medical University, Dalian City, Liaoning Province, China.

**Keywords:** pathology, thyroid cancer, ultrasound

## Abstract

**Background:**

In recent years, the incidence rate of thyroid cancer is increasing. Meanwhile, with the development of medical technology, the detection rate of thyroid cancer by ultrasound has been greatly improved. Normally doctors can initially distinguish pathology of malignant thyroid diseases by their abundant experience. And it will bring assistance to follow-up treatment. However, the results of these studies have been contradictory. Therefore, this meta-analysis tested the hypothesis that ultrasound is accurate in distinguishing pathology of malignant thyroid diseases.

**Methods:**

We will search PubMed, Web of Science, Cochrane Library, and Chinese biomedical databases from their inceptions to the December 14, 2021, without language restrictions. Two authors will independently carry out searching literature records, scanning titles and abstracts, full texts, collecting data, and assessing risk of bias. Review Manager 5.2 and Stata14.0 software will be used for data analysis.

**Results:**

This systematic review will determine the accuracy of ultrasound in distinguishing pathology of malignant thyroid diseases.

**Conclusion:**

It is findings will provide helpful evidence for the accuracy of ultrasound in distinguishing pathology of malignant thyroid diseases.

**Systematic review registration:**

INPLASY2021120072.

## 1 Introduction

Thyroid cancer is a common tumor, accounting for about 1.3% of all malignant tumors. High resolution ultrasound imaging technology can not only detect thyroid space occupying lesions earlier but also can clearly show thyroid's structures and the number, size and shape of lesions. What is more, ultrasound has no radiation, convenient and shorter waiting time, which makes it more popular than X-ray or CT in diagnosing thyroid diseases. Therefore, ultrasound has become the first imaging method for diagnosing thyroid cancer.^[1,2]^ At the same time, a great number of studies have confirmed that ultrasound can successfully distinguish thyroid papillary carcinoma and thyroid follicular carcinoma. Although, the diagnostic rate of medullary carcinoma is lower, it still has statistical significance.^[3,4]^ Though the five-year survival rate of thyroid cancer can reach about 60% to 90%, if we can detect pathology of the lesions, it have great significance to prolong patients’ life.^[5]^ With the development of ultrasound, ultrasound can detect that different pathological thyroid cancers have diverse image characteristics.^[6]^ Meanwhile, large number of studies have confirmed that ultrasound has high effectiveness and reliability in distinguishing pathology of malignant thyroid diseases.

## 2 Materials and methods

This study was conducted in accordance with the Preferred Reporting Items for Systematic Reviews and Meta-Analyses guidelines and the protocol was registered in the INPLASY (INPLASY2021120072).

### 2.1 Eligibility criteria

#### 2.1.1 Type of study

This study will only include high quality clinical cohort or case control studies.

#### 2.1.2 Type of patients

The patients should be those who had undergone thyroid cancer.

#### 2.1.3 Intervention and comparison

This study compares ultrasound with pathology for distinguishing pathology of malignant thyroid diseases.

#### 2.1.4 Type of outcomes

The primary outcomes include sensitivity, specificity, positive and negative likelihood ratio, diagnostic odds ratio, and the area under the curve of the summary receiver operating characteristic.

### 2.2 Search methods

PubMed, Web of Science, Cochrane Library, and Chinese biomedical databases will be searched from their inceptions to the December 14, 2021, without language restrictions. The search strategy for PubMed is shown in Table 1. Other online databases will be used in the same strategy.

**Table 1:** Search strategy sample of pubmed.

Number	Search terms
1	Thyroid cancer or thyroid tumors or malignant thyroid diseases
2	Ultrasound or ultrasonography or ultrasonic diagnos
3	Pathology
4	and 1–3

### 2.3 Data extraction and quality assessment

Two authors will independently select the trials according to the inclusion criteria, and import into Endnote X9 (Thomson Corporation, Stanford, USA). Then remove duplicated or ineligible studies. Screen the titles, abstracts, and full texts of all literature to identify eligible studies. All essential data will be extracted using previously created data collection sheet by 2 independent authors. Discrepancies in data collection between 2 authors will be settled down through discussion with the help of another author. The following data will be extracted from each included research: the first authors surname, publication year, language of publication, study design, sample size, number of lesions, source of the subjects, instrument, “gold standard,” and diagnostic accuracy. The true positives, true negatives, false positives, and false negatives in the fourfold (2 × 2) tables were also collected. Methodological quality was independently assessed by 2 researchers based on the quality assessment of studies of diagnostic accuracy studies (QUADAS) tool. The QUADAS criteria included 14 assessment items. Each of these items was scored as “yes” (2), “no” (0), or “unclear” (1). The QUADAS score ranged from 0 to 28, and a score ≥22 indicated good quality. Any disagreements between 2 investigators will be solved through discussion or consultation by a 3rd investigator.

### 2.4 Statistical analysis

The STATA version 14.0 (Stata Corp, College Station, TX, USA) and Meta-Disc version 1.4 (Universidad Complutense, Madrid, Spain) softwares were used for meta-analysis. We calculated the pooled summary statistics for sensitivity, specificity, positive and negative likelihood ratio, and diagnostic odds ratio with their 95% confidence intervals. The summary receiver operating characteristic curve and corresponding area under the curve were obtained. The threshold effect was assessed using Spearman correlation coefficients. The Cochran's Q-statistic and I test were used to evaluate potential heterogeneity between studies. If significant heterogeneity was detected (*Q* test *P* < .05 or *I* test > 50%), a random effects model or fixed effects model was used. We also performed sub group and meta-regression analyses to investigate potential sources of heterogeneity. To evaluate the influence of single studies on the overall estimate, a sensitivity analysis was performed. We conducted Begg funnel plots and Egger linear regression tests to investigate publication bias.

### 2.5 Ethics and dissemination

We will not obtain ethic documents because this study will be conducted based on the data of published literature. We expect to publish this study on a peer-reviewed journal.

## 3 Discussion

By detecting size, number, shape, boundary, blood flow, internal echo and calcification features of malignant lesions, ultrasound can help doctors distinguish pathology of cancer without pathological diagnosis, which can bring patients less pain. At the same time, ultrasound will bring help for the patients’ prognosis and further treatments.^[7]^ It has high specificity in distinguishing pathology of malignant thyroid diseases, and will become an important auxiliary tool for thyroid disease diagnosis.

## Author contributions

**Conceptualization:** Lipeng Sun, Hui Wang.

**Data curation:** Ling Qin, Qiyu Liu.

**Methodology:** Ling Qin, Qiyu Liu.

**Writing – original draft:** Ling Qin.

**Writing – review & editing:** Ling Qin, Hui Wang.
